# Suppression of cerebral ischemia injury induced blood brain barrier breakdown by dexmedetomidine via promoting CCN1

**DOI:** 10.18632/aging.205557

**Published:** 2024-02-15

**Authors:** Shuangmei Liu, Xuepeng Jia, Bo Liu, Yue Liu, Hong Yin

**Affiliations:** 1 Department of Anesthesiology, Shengjing Hospital of China Medical University, Shenyang 110004, Liaoning, China; 2 Medical Research Center, Shengjing Hospital of China Medical University, Shenyang 110004, Liaoning, China; 3 Liaoning Key Laboratory of Research and Application of Animal Models for Environmental and Metabolic Diseases, Shenyang 110004, Liaoning, China

**Keywords:** dexmedetomidine, cerebral ischemia injury, blood brain barrier, CCN1, middle cerebral artery occlusion

## Abstract

Background: Blood-brain barrier (BBB) could aggravate cerebral ischemia injury. Dexmedetomidine (Dex) has been believed to play a protective role in cerebral ischemia injury-induced BBB injury.

Methods: Middle cerebral artery occlusion (MCAO) and oxygen-glucose deprivation (OGD) models were established to simulate cerebral ischemia injury. Animal experiments included 4 groups, Sham, MCAO, MCAO+Dex, MCAO+Dex+sh-CCN1. Generally applicable gene set enrichment analysis was performed to analyze gene expression difference. Total collagen content and Evans blue staining were performed to measure infarct ratio and BBB breakdown, respectively. The cell apoptosis, mRNA and protein expression were measured through flow cytometry, PCR, and western blotting, respectively. The levels of IL-1β, TNF-α, and IL-6 in serum were measured with commercial ELISA kits.

Results: Dex greatly promoted the expression level of CCN1. Dex suppressed cerebral ischemia injury, increased tight junction protein expression, improved the memory ability and neurological function of MCAO rats through targeting CCN1. The significant increase of inflammatory factors in the serum of MCAO rats were suppressed by Dex. Dex suppressed OGD induced increase of HRP permeability and promoting tight junction protein expression *in vitro* through regulating CCN1. The neurological function evaluation was performed with Neurological Severity Score (NSS) and Longa Score Scale.

Conclusions: Dex could remarkably alleviate cerebral ischemia injury by inhibiting BBB breakdown, inflammatory response, and promoting neurological function and tight junction protein expression via up-regulating CCN1. This study might provide a novel therapeutic target for the prevention and treatment of cerebral ischemia injury-induced BBB.

## INTRODUCTION

Cerebral ischemia injury is characterized by high morbidity and mortality. At present, the most effective treatment is intravenous thrombolysis and intravascular therapy [1]. Timely recovery of blood flow reperfusion can prevent irreversible brain tissue damage. However, the treatment window is narrow after stroke onset, and delayed thrombolytic therapy will lead to serious complications, including cerebral hemorrhage and edema [2, 3]. Seeking novel therapeutic strategies is necessary for the prevention and treatment of cerebral ischemia injury.

Blood-brain barrier (BBB) exists between brain tissue and blood, which can effectively regulate the material transport and metabolism on both sides of the barrier, thus ensuring the relative stability of the internal environment of central nervous tissue [4, 5]. BBB is composed of cerebral vascular endothelial cells, astrocytes, and matrix. The structural and functional integrity of BBB is damaged after cerebral ischemia injury, which not only affects thrombolytic therapy, but also aggravates brain injury after reperfusion [6, 7]. Therefore, protecting BBB on the basis of thrombolysis or thrombectomy becomes one of the important therapeutic strategies to improve the prognosis of cerebral ischemia injury.

Dexmedetomidine (Dex) is a highly selective α2-adrenergic receptor agonist, with central anti sympathetic effect, can produce sedative, analgesic, and anti-anxiety effects [8, 9]. Dex has no obvious inhibitory effect on respiration, and it is widely used in clinical practice [10]. Dex may play an organ protective role by stabilizing sympathetic nervous system, regulating transmitter release, inhibiting oxidative stress response, affecting inflammatory response, activating cell protective signal pathway, and regulating cell apoptosis [11–13]. The protection role of Dex in cerebral ischemia injury-induced BBB has been reported previously, but the specific regulatory mechanism has not been fully clarified.

CCN1, also known as CYR61 (cysteine rich protein 61), is a 40 kDa exocrine protein and belongs to CCN family [14]. Previous studies have shown that CCN protein family members play an important role in cell adhesion, migration, proliferation, differentiation and other processes, and participate in important biological processes such as angiogenesis and wound healing [15]. If CCN1 is involved in the regulation of Dex in cerebral ischemia injury-induced BBB has not been reported.

In this study, middle cerebral artery occlusion (MCAO) and oxygen-glucose deprivation (OGD) models were established to simulate cerebral ischemia injury. Generally applicable gene set enrichment analysis was performed to analyze gene expression difference. Morris water maze test, neurological Severity Score and Longa Score Scale were performed to evaluate neurological function of animals. We firstly demonstrated that Dex significantly improved cerebral ischemia injury via suppressing BBB breakdown, inflammatory response, and promoting neurological function and tight junction protein expression. This study might provide a novel therapeutic strategy for the prevention and treatment of cerebral ischemia injury.

## MATERIALS AND METHODS

### Cell culture

Human brain microvascular endothelial cells (HBMEC) were purchased from Chinese Academy of Science (Beijing, China). DMEM (Dulbecco’s modified Eagle medium, #12491015, Gibco, USA) and 10% FBS (#A5669501, Gibco) were used for cell culture at 5% CO_2_ and 37°C.

### OGD treatment

The cells were cultured with DMEM containing 1% penicillin/streptomycin and 10% fetal bovine serum. The cells were incubated on the condition of 37°C and 5% CO_2_. The cells were treated with Dex (50 μM) or sh-CCN1 (20 nM) for 24 h. Then, OGD was performed to treat cells. OGD was established with the condition of 2% oxygen, 93% N_2_, and 5% CO_2_ for 4 h in the 37°C chamber. Then, re-oxygenation was performed for 12 h at 37°C with 5% CO_2_. Finally, the cells were used for experiments.

### Flow cytometry

Cells were firstly plated into 6-well plates, and cultured on the condition of 5% CO_2_ and 37°C. The cells in different groups were treated as described in the part 2.2. After digestion, the cell pellets were collected and suspended with cold PBS containing propidium iodide and Annexin V-FITC (#C1062S, Beyotime, China). After incubation in the dark for 15 min, cell apoptosis was measured using flow cytometry.

### RT-PCR

Trizol (#R0016, Beyotime) method was used to extract RNA from tissues. After RNA extraction, Nanodrop 2000 spectrophotometer (Thermo Fisher Scientific, USA) was used to determine the concentration and purity of RNA. The purity was 1.8–2.0 measured through D260/D280 method, and reverse transcription was performed. Reverse transcription was performed with Takara PrimeScript RT reagent kit with gDNA eraser kit (#RR047A) according to the instructions. Sybgreen method was used for qRT-PCR. Step one plus from ABI company (USA) was used for PCR, β-actin was used as the internal reference gene, and the relative expression level of gene was measured through 2^−ΔΔCT^ method. The primers of CCN1 and β-actin are listed as follows: CCN1 (F: GGTCAAAGTTACCGGGCAGT, R: GGAGGCATCGAATCCCA), β-actin (F: TGGCACCCAGCACAATGAA, R: CTAAGTCATAGTCCGCCTAGAAGCA).

### Western blotting

Cells were firstly lysed using lysis buffer (Nanjing Jiancheng, Beijing, China). The protein concentrations were measured using Pierce™ 660 nm reagent (#22660, Thermo Fisher Scientific, USA). Same amount of protein (20 μg) was loaded for 10% SDS-PAGE, and transferred to a PVDF membrane. TBST containing 5% non-fat milk was used to block membrane for 2 h. Then, the proteins were incubated with related primary antibodies (Rabbit polyclonal to CCN1, ab230947; Rabbit monoclonal to GAPDH, ab9485) at 4°C overnight. After washing with PBS, the membrane was incubated with secondary antibodies for 2 h. Then, membrane was measured using an enhanced chemiluminescence detection kit (#34580, Thermo Fisher Scientific, USA), and ImageJ software was used to analyze protein band.

### Generally applicable gene set enrichment analysis

We used DESeq2 to perform differential expression analysis between groups. DESeq2 package requires “raw” counts of sequencing reads as the starting point for differential expression analysis. Therefore, before submitting to the program for analysis, the count matrix was not normalized, which is explicitly required by the software. We ran the “DESeq Data Set From Matrix” function, with the count matrix for all samples and the design matrix as input data, which produced an R object for the downstream differential expression analysis. Including count data for all the samples is more robust for estimating parameters. For differential expression analysis between a certain group, we used “contrast” function. The result file that contains *p* values and fold changes for each gene was generated with the “results” function [16].

The log2 fold changes for all the genes from the differential expression analysis were submitted as an input file for GAGE analysis [17]. GAGE then uses the information of fold change for each gene to obtain mean and standard deviation of fold changes for a gene set (pathway) as well as for the background (the whole transcriptome) and generate a *t* test statistic and *p* value for a comparison in fold change between a gene set and the background. Essentially, if there is significant difference in fold change between a gene set and the background, an extreme *t* statistic and a small *p* value will be achieved. As the fold change involves information on up- or downregulation, a pathway identified by GAGE will also be indicated as up- or downregulated. As a nice feature of GAGE, a KEGG pathway identified as significantly differentially expressed can be visualized in a KEGG pathway plot [18].

### MCAO experiment

All animal experiments were approved by Medical Ethics Committee of Shengjing Hospital affiliated to China Medical University (2015PS122k), in accordance with the animal care guidelines of NIH (USA). Male SD rats (230–250 g) purchased from Charles River Laboratories (Beijing, China) were used in this study. The animals were raised on the condition of 40–60% humidity, 24–27°C. Animals were divided into 4 groups randomly with 5 animals in each group. The animals were anesthetized with intramuscular injection of ketamine (60 mg/ml) and xylazine (10 mg/ml). Left side occlusion of the middle cerebral artery with silicone-coated sutures was performed to establish MCAO model. After occlusion for 2 h, reperfusion was performed. The rats in the group MCAO+Dex were injected with Dex (9 μg/kg) through tail vein at the onset of reperfusion for 1 h. The rats in the group MCAO+Dex+sh-CCN1 were injected with Dex (9 μg/kg) and sh-CCN1 vectors (1 × 10^8^ transfection units/mL) through tail vein at the onset of reperfusion for 1 h. The animals in the group Sham and MCAO were injected with same amount of normal saline. The neurological function evaluation and Morris water maze test was performed 24 h after MCAO induction. Then, the animals were sacrificed, and brain tissues were collected.

### Morris water maze test

A white circular pool with 300 cm diameter was used in this study. The pool was filled with water, and four equal quadrants were divided. The starting locations were set in the 4 quadrants, a 2 cm-escape platform was set. Nontoxic white paint was used to camouflage this platform. The positions of animals were recorded with a camera and computer assisted tracking system (Shanghai Ruanlong Science and Technology Development, China). The animals were put into the water tank from the water surface to the pool wall. The time required for the animals from entering the water to climbing the platform was recorded (Escape latency), and the swim distance was analyzed.

### Immunofluorescence staining

Paraffin was used to embed tissues. Antigen repair was performed after dewaxing. 3% hydrogen peroxide was added to the slices to block the endogenous peroxidase. The slices were incubated at room temperature for 15 min with 3% hydrogen peroxide and washed with PBS three times (3 min/time). After blocking with serum, the sections were incubated with the primary antibody. After 3 times washing with PBS, the second antibody was added to incubate sections (2 h). Then, DAB chromogenic solution was added. After dehydration and mount, the sections were captured with Olympus BX41 microscope (Tokyo, Japan). Two pathologists are required to evaluate the immunohistochemical staining results.

### TTC (2,3,5-Triphenyl tetrazolium chloride) staining

The brain was collected after anesthesia, and then frozen at −20°C for 20 minutes. The first cut was at the midpoint of the line between the anterior pole of the brain and the optic chiasma with 2 mm thickness section. The second cut was at the optic chiasma. The third cut was at the funnel handle. The fourth cut was between the funnel handle and the caudal pole of the posterior leaf. 2% TTC solution (#17779, Millipore, USA) were used to stain brain tissues for 20 min in the dark. The tissue slices were flipped occasionally to ensure that the tissues were immersed in the staining solution. The tissues were fixed with formalin for 12 h at 4°C. Then, the stained tissues were captured with a digital camera, and cerebral infarction size was quantified with Image Pro-Plus software.

### Evans blue staining

The integrity of the blood barrier was assessed by Evans blue method. At 48 hours after MCAO, Evans blue dye (E2129, 2%, 5 mL/kg, Sigma Aldrich, USA) was injected into the right femoral vein with a needle. Once injected, remove the needle and press the injection site with cotton to stop bleeding. After 60 minutes, the animals were anesthetized with intramuscular injection of ketamine (60 mg/ml) and xylazine (10 mg/ml), and perfused with normal saline through the heart to remove the dye in the blood vessels. Brain samples were weighed and homogenized in 2 ml of 50% trichloroacetic acid (T6399, Sigma Aldrich), incubated overnight at 4°C, and centrifuged at 13000 g for 30 minutes. The amount of Evans blue in the supernatant was quantified at 620 nm by spectrophotometry. The results are expressed in micrograms per gram of wet weight of brain tissue.

### Inflammatory factor detection

The levels of IL-1β (#H002-1-2), TNF-α (#H052-1-2), and IL-6 (#H007-1-2) were measured with related commercial kits purchased from Nanjing Jiancheng Bioengineering Institute according to instructions.

### Neurological function evaluation

Neurological Severity Score (NSS) and Longa Score Scale were performed to evaluate neurological function of animals as described previously [19].

### Horseradish peroxidase (HRP) permeability measurement

The cells (3 × 10^4^) were evenly spread in the upper chamber, and the complete cell culture medium with equal liquid level was added in the lower chamber, and cultured in the incubator. OGD modeling was carried out when the cells fully adhered to the wall and grew into monolayer cells, and the cells were treated with Dex or sh-CCN1 as described in part 2.2. After cell re-oxygenation, HRP (50 nmol/L, #M027, Nanjing Jiancheng Bioengineering Institute, China) was added to the upper chamber, and the chamber liquid was removed 1 h later. The absorbance was detected at 450 nm to calculate the concentration of HPR.

### High throughput sequencing of mRNA

Total RNA from the samples were extracted with TRIzol® Reagent (Invitrogen, USA), and Nanodrop 2000 was used to detect the concentration and purity of the extracted RNA. The RNA integrity was detected with agarose gel electrophoresis, and RIN value was determined with Agilent 2100. The total amount of RNA required for single database establishment was >5 ug, and the concentration was ≥200 ng/μL. OD260/280 was between 1.8–2.2. Then, Ribo Zero Magnetic Kit (EpiCentre) was used to remove rRNA, RNase R (EpiCentre) was used to remove linear RNA, TruSeqTM Stranded Total RNA Library Prep Kit (Illuminata) was used to construct Paired End sequencing library, and Hiseq4000 sequencing platform was used for sequencing analysis. SepPrep and Sickle software were used to detect the data quality of offline data. The data obtained after quality control were compared and analyzed with reference genomic human data using Bowtie.

### Statistical analysis

SPSS19.0 software was used for statistical analysis. All experiments were repeated at least 3 times, and the quantitative results were expressed by mean ± standard deviation. Single factor analysis of variance (ANOVA) was used to compare the quantitative values between multiple groups, and LSD method was used to compare the two groups. Inspection level α = 0.05, and Image-pro J software is used for gray value analysis.

### Availability of data and material

The data and material used to support the findings of this study are included within the manuscript and supplementary files.

## RESULTS

### Dex greatly promoted the expression level of CCN1

**Figure 1:**
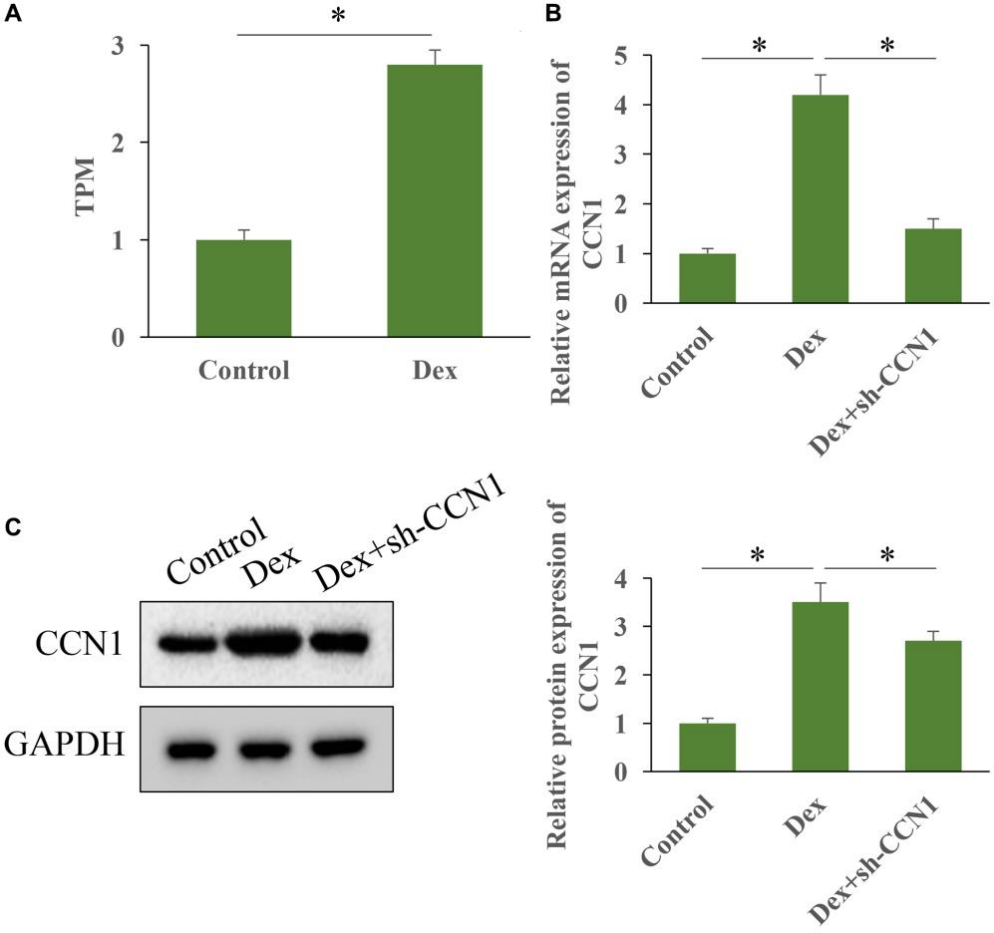
**Dex greatly promoted the expression level of CCN1.** (**A**) The level of CCN1 in HBMEC was measured with high throughput sequencing; (**B**) The level of CCN1 was measured with RT-PCR; (**C**) The level of CCN1 was measured with western blot. *n* = 3.

High throughput sequencing was performed to investigate the influence of Dex treatment on expression of CCN1 (Figure 1A). We found that Dex treatment markedly increased the level of CCN1 *in vivo* (Figure 1A). In addition, the mRNA and protein expression intensity of CCN1 was markedly elevated by Dex, but transfection with sh-CCN1 significantly suppressed the level of CCN1 (Figure 1B, 1C).

### Dex suppressed cerebral ischemia injury and increased tight junction protein expression

**Figure 2:**
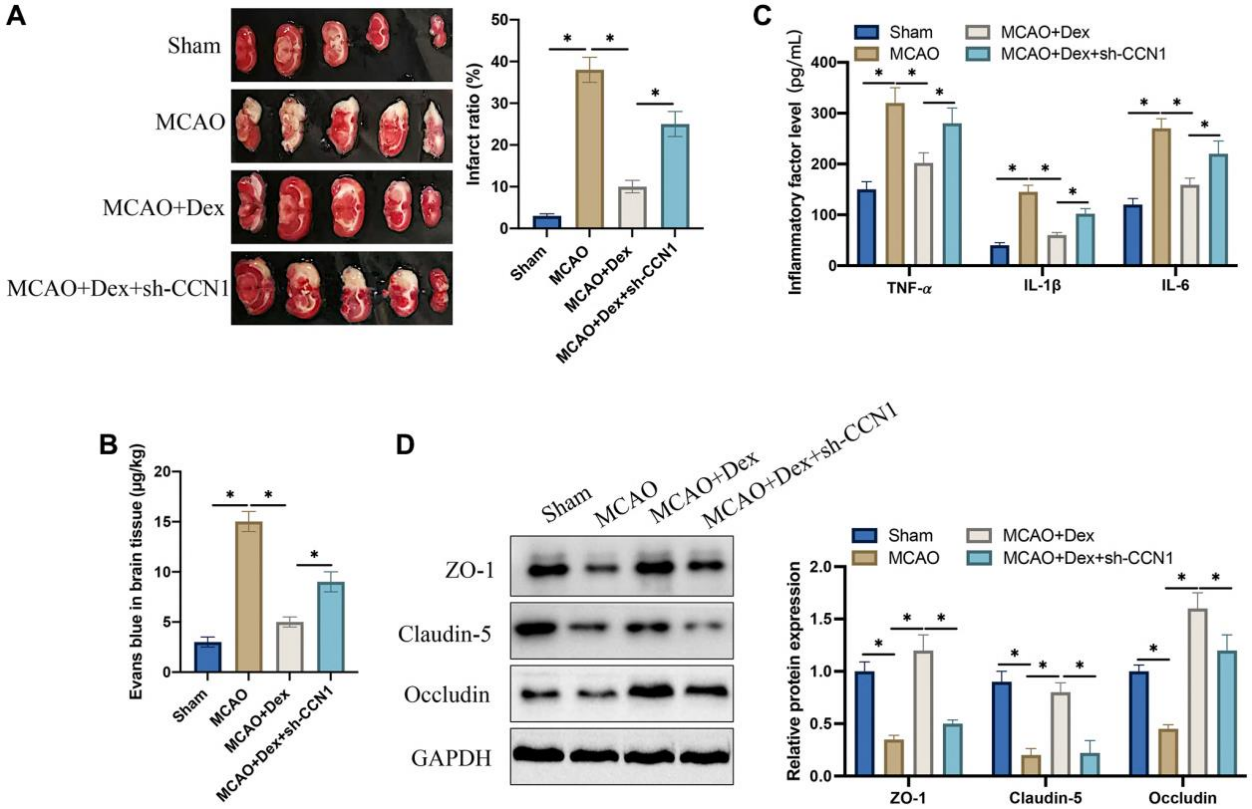
**Dex suppressed cerebral ischemia injury and increased tight junction protein expression.** (**A**) TTC staining was performed to study the infarct ratio; (**B**) Evans blue staining was conducted to investigate blood brain barrier injury; (**C**) The levels of IL-6, IL-1β, and TNF-α in serum were detected; (**D**) The expression levels of tight junction proteins were determined in brain tissues. ^*^p <0.05. *n* = 5.

The MCAO animal model was firstly established. We found that the remarkable increase of infarct ratio in MCAO rats was greatly inhibited after Dex treatment (Figure 2A). However, the infarct area was elevated greatly by treatment with sh-CCN1 vectors (Figure 2A). The BBB breakdown *in vivo* was assessed through Evans blue staining. The blood brain barrier breakdown was aggravated in the MCAO model, but it was relieved after Dex treatment (Figure 2B). However, knockdown of CCN1 could aggravate the blood brain barrier breakdown. In addition, we found that Dex administration could decrease the levels of inflammatory factors including IL-6, IL-1β, and TNF-α (Figure 2C), but they were restrained after knocking down CCN1 (Figure 2C). We also investigate the levels of tight junction protein expression with Immunofluorescence staining after Dex or sh-CCN1 treatments. Dex could markedly promote the levels of Occludin, Claudin-5, and ZO-1, but they were greatly suppressed by sh-CCN1 (Figure 2D). These finding indicate that Dex might regulate cerebral ischemia injury induced blood brain barrier breakdown through regulating CCN1.

### Dex remarkably improved the memory ability and neurological function of MCAO rats

**Figure 3:**
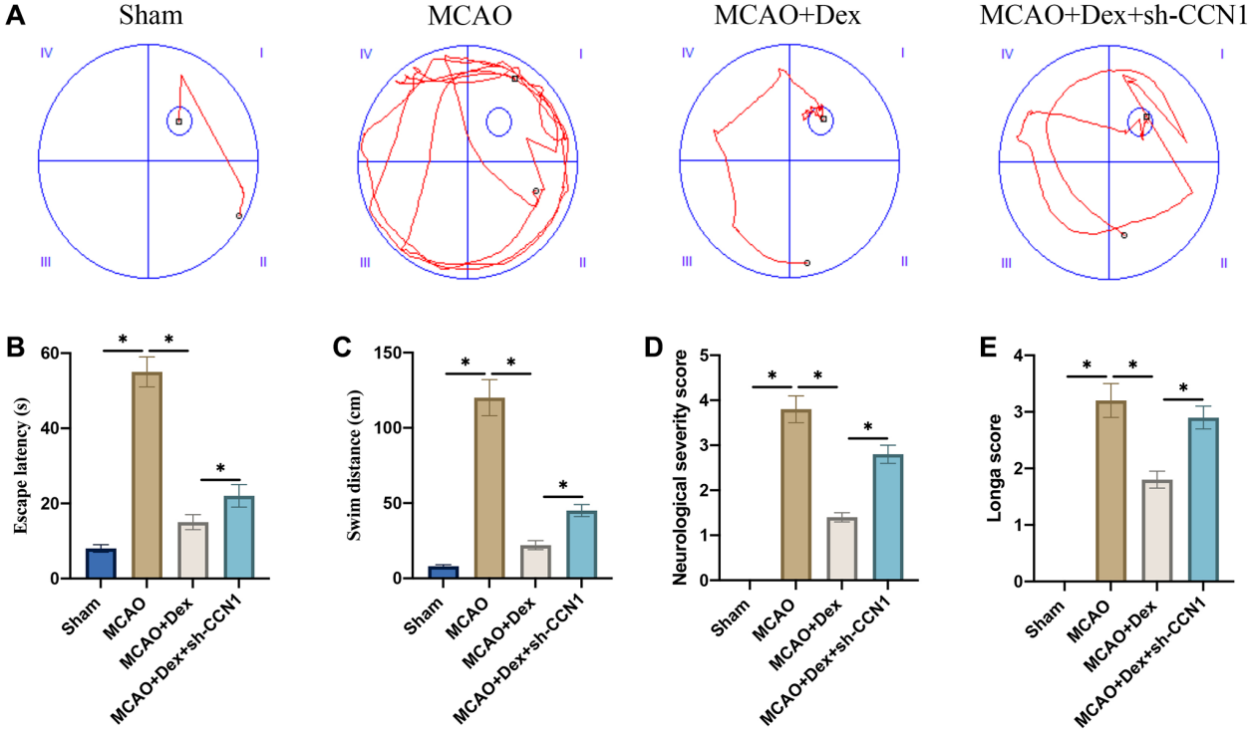
**Dex remarkably improved the memory ability and neurological function of MCAO rats.** (**A**) Morris water maze test was performed to evaluate the influence of Dex and sh-CCN1 on memory ability of rats (*n* = 5); (**B**) Escape latency was analyzed; (**C**) Swim distance was calculated; (**D**) The neurological function was evaluated with neurological severity score; (**E**) The neurological function was evaluated with Longa score. ^*^p < 0.05. *n* = 5.

Morris water maze test was performed to evaluate the influence of Dex and sh-CCN1 on memory ability of rats. Significant increase of escape latency and swim distance were observed in the group MCAO (Figure 3A–3C). However, Dex treatment significantly inhibited escape latency and swim distance compared with group MCAO (Figure 3A–3C), but the change trends of escape latency and swim distance induced by Dex were greatly reversed by sh-CCN1. The neurological function was evaluated with neurological severity and Longa scores (Figure 3D, 3E). The remarkable improvement of neurological function caused by Dex was markedly inhibited by sh-CCN1 (Figure 3D, 3E).

### Gene differences, gene ontology, and pathway map analysis

**Figure 4:**
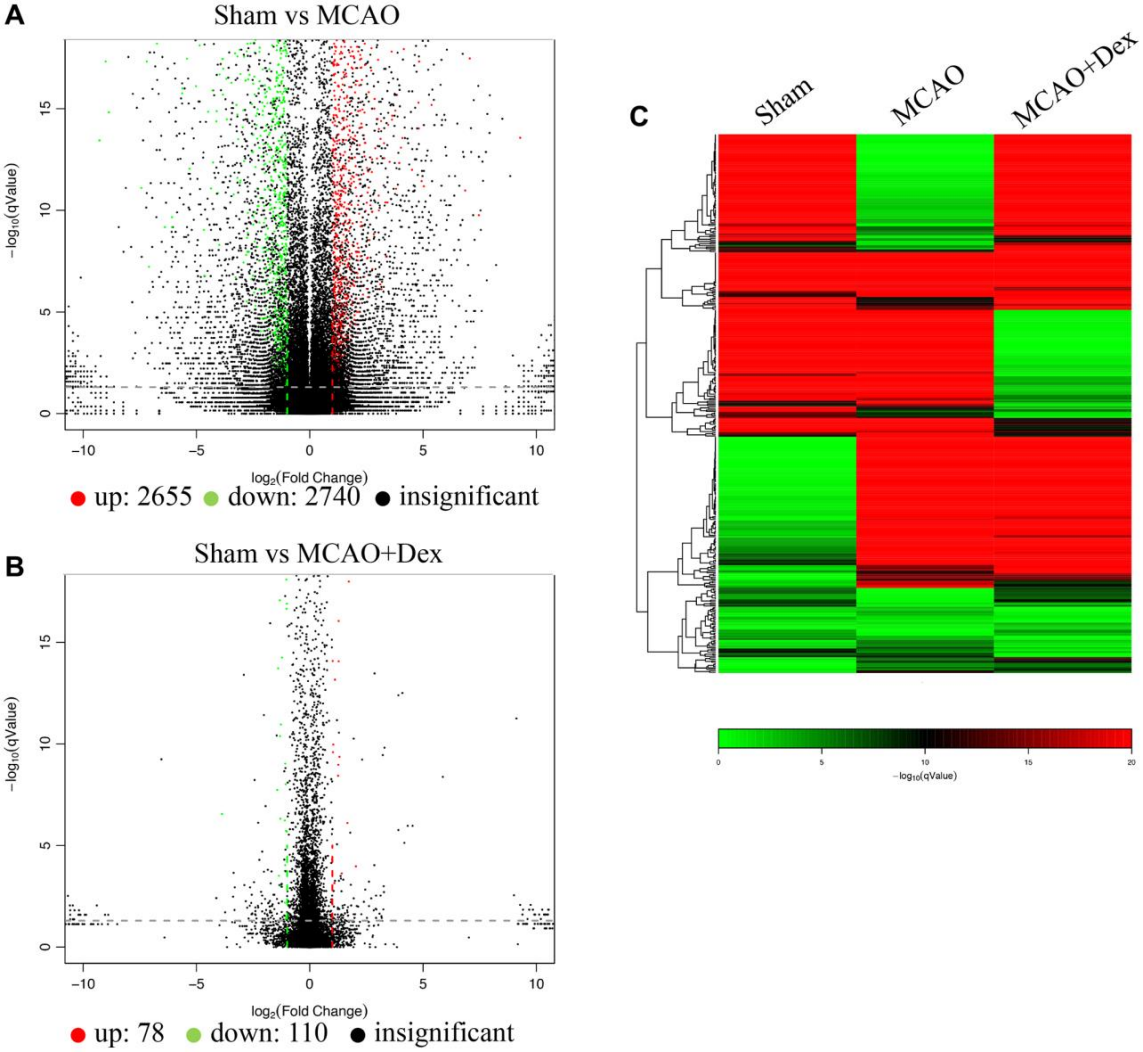
**Gene differences analysis using brain tissues.** (**A**) Gene differences between group Sham and MCAO were analyzed using volcano plot; (**B**) Gene differences between group Sham and MCAO+Dex were analyzed using volcano plot; (**C**) Gene differences were analyzed using Pheatmap analysis. *n* = 3.

Gene differences were analyzed using volcano plot (Figure 4A, 4B), which indicate 2655 up-regulated genes and 2740 down-regulated genes between group Sham and group MCAO. However, 78 up-regulated genes and 110 down-regulated genes between group Sham and group MCAO+Dex were observed, which suggests that Dex treatment reduced the gene difference changes. Similar data was obtained through Pheatmap analysis (Figure 4C, red color: up-regulated genes, green color: down-regulated genes, black color: un-different genes).

**Figure 5:**
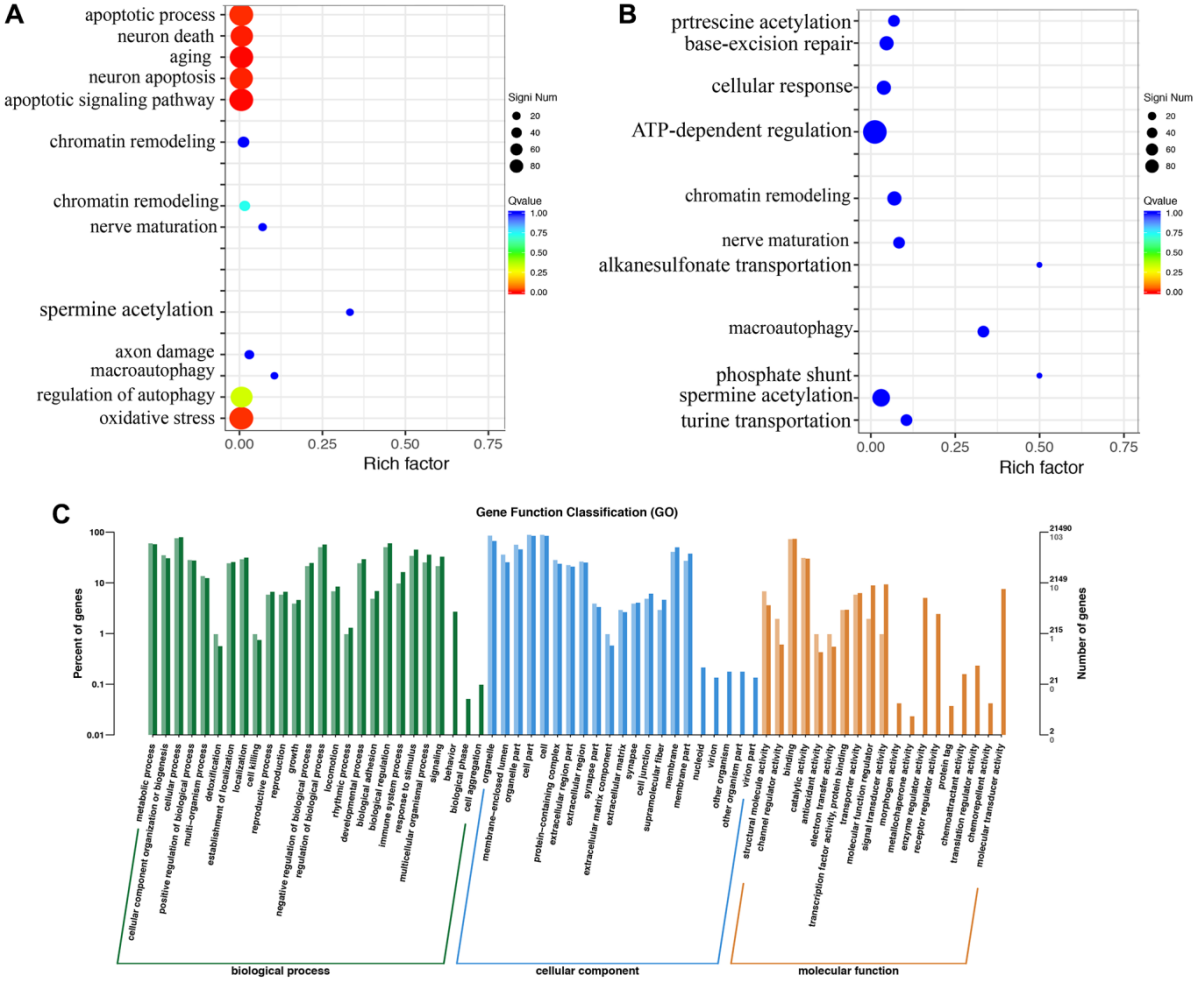
**Gene ontology enrichment analysis using brain tissues.** (**A**) Gene ontology pathway enrichment analysis in the group MCAO; (**B**) Gene ontology pathway enrichment analysis in the group MCAO+Dex; (**C**) Differential gene GO annotation classification histogram. *n* = 3.

The gene ontology enrichment analysis indicated that MCAO treatment might affect the apoptotic process, neuron death, aging, and oxidative stress (Figure 5A). However, Dex treatment changed the influence of MCAO (Figure 5B). The gene ontology analysis includes molecular function, cellular component and biological process (Figure 5C).

**Figure 6:**
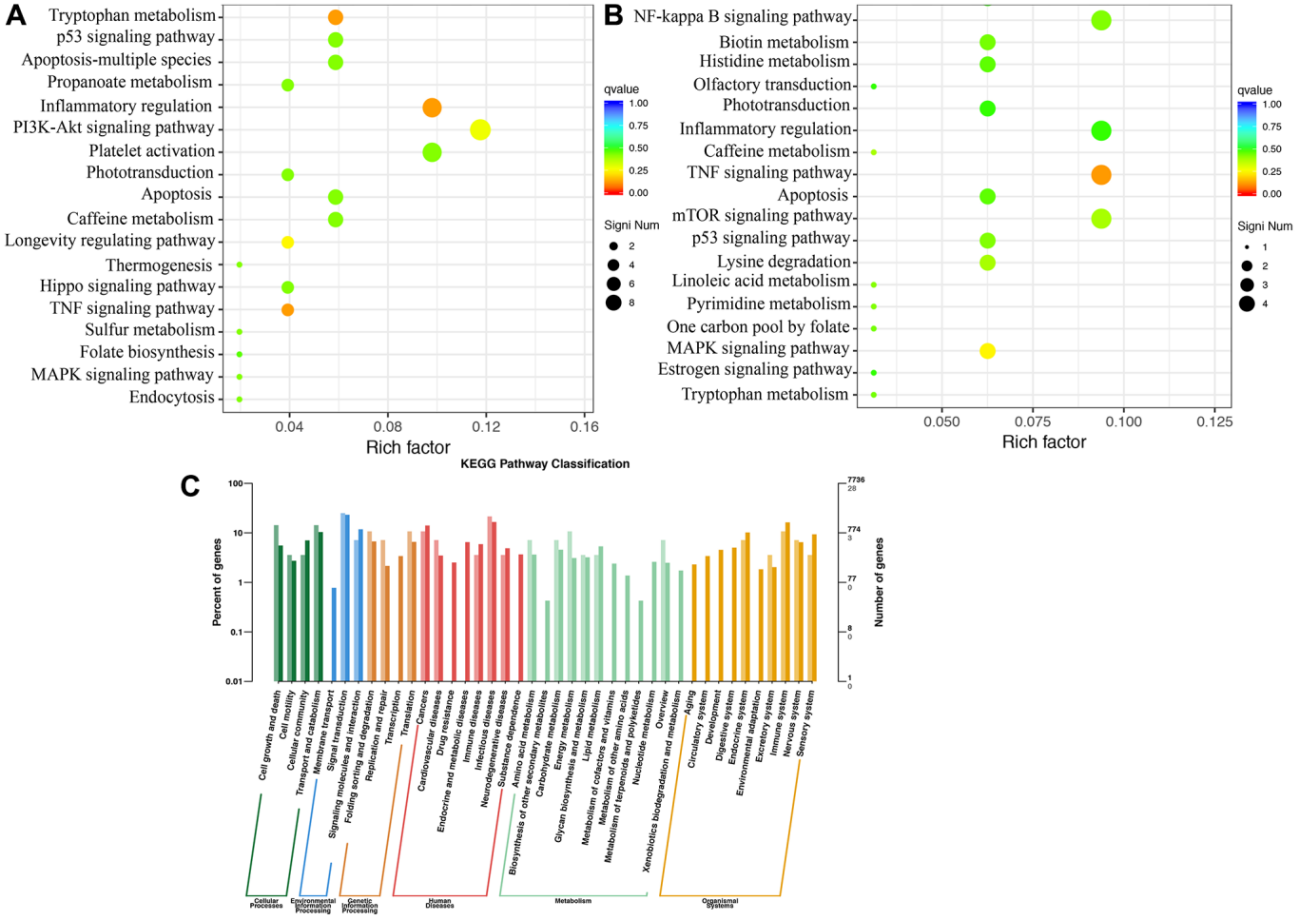
**KEGG pathway enrichment analysis using brain tissues.** (**A**) KEGG pathway enrichment analysis in the group MCAO; (**B**) KEGG pathway enrichment analysis in the group MCAO+Dex; (**C**) KEGG pathway classification was analyzed. *n* = 3.

The KEGG pathway enrichment analysis suggested that inflammatory regulation, PI3K-Akt signaling pathway, apoptosis, and TNF signaling pathway are the most critical pathways involved in MCAO rats (Figure 6A). Compared to group MCAO, differential signaling pathway influenced by Dex were mainly associated TNF signaling pathway, inflammatory regulation, mTOR signaling pathway, and NF-kappa B signaling pathway (Figure 6B). In addition, the KEGG pathway classification was presented (Figure 6C).

### Dex suppressed OGD induced increase of HRP permeability and promoting tight junction protein expression *in vitro*

**Figure 7:**
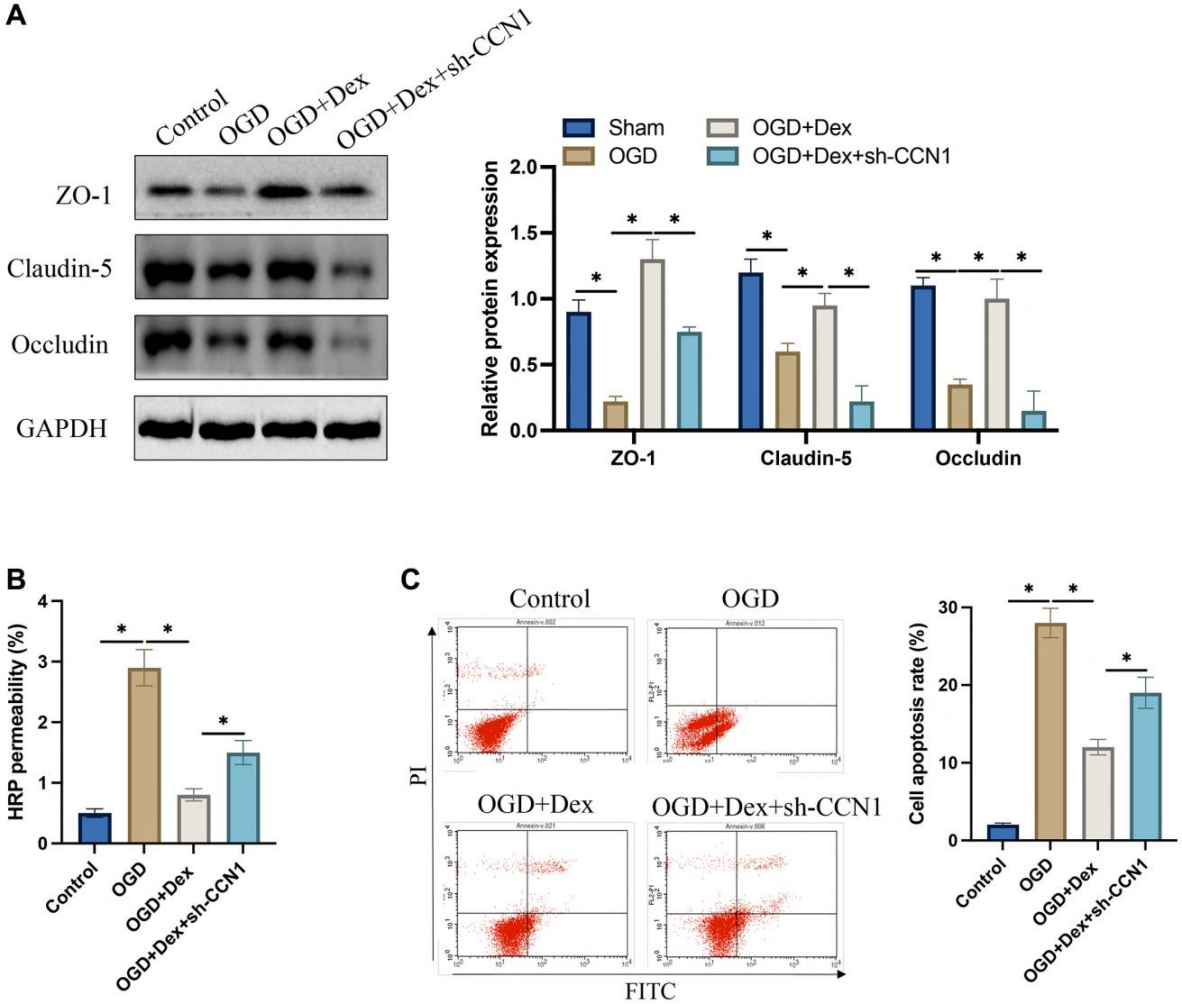
**Dex suppressed OGD induced increase of HRP permeability and promoting tight junction protein expression *in vitro*.** (**A**) The expression levels of tight junction proteins in HBMEC were measured; (**B**) Blood brain barrier *in vitro* was evaluated with HRP permeability; (**C**) Cell apoptosis was detected with flow cytometry. ^*^p < 0.05. *n* = 3.

The protein expression of Occludin, Claudin-5, and ZO-1 was markedly suppressed after OGD induction. After treatment with Dex, the expression of tight junction protein in cells were greatly elevated, but sh-CCN1 reversed the influence of Dex (Figure 7A). The increase of HRP permeability induced by OGD was significantly inhibited by Dex (Figure 7B), but the decreased HRP permeability was increased after simultaneous transfection with sh-CCN1. In addition, we found that transfection with sh-CCN1 could increase the decreased apoptosis caused by Dex (Figure 7C).

## DISCUSSION

After the restoration of blood perfusion, the ischemic tissues will undergo a pathological process that further aggravates the functional and metabolic disorders of tissues and cells and structural damage, which is called ischemia reperfusion injury [20, 21]. In clinic, ischemia and hypoxia of organs and tissues caused by various reasons will involve ischemia reperfusion injury in the process of treatment and recovery, such as organ transplantation, brain stroke and traumatic shock [22, 23]. In this study, MCAO and OGD models could simulate ischemia reperfusion injury *in vivo* and *in vitro* levels, respectively.

BBB is commonly observed after cerebral ischemia injury, and BBB could further aggravate brain injury [24]. With the increase of BBB permeability, a large amount of albumin in the blood is secreted to the ipsilateral BBB, causing intracranial hypertension to aggravate brain edema and acute intracranial hemorrhage after thrombolysis [25, 26]. The increase of BBB permeability will obviously aggravate the damage of ischemic brain tissue. Many research results show that the changes of BBB function and structure are an important pathophysiological process in the early stage of cerebral ischemia reperfusion [27]. Tight junction protein is composed of three membrane proteins and closed small loop proteins. Tight junction proteins are the basis of BBB integrity [1, 2]. The destruction of tight junction protein is the main cause of BBB destruction after ischemic stroke [28]. In the present study, the increased tight junction proteins after MCAO were suppressed by Dex, and the influence of Dex on tight junction protein was reversed by sh-CCN1.

After cerebral ischemia injury, infiltration and aggregation of peripheral immune cells and molecules into brain parenchyma are considered to be the causes of BBB dysfunction and damage progression [3, 23]. On the one hand, BBB is destroyed after cerebral ischemia, white blood cells are recruited and infiltrated, causing inflammatory cascade reaction. On the other hand, pro-inflammatory factors such as cytokines produced by inflammatory reaction further destroy BBB [29, 30]. Activation of microglia and astrocytes caused by ischemia leads to production of pro-inflammatory cytokines (IL-6, IL-1β, and TNF-α), and the release of chemotactic factors, which further increase the permeability of BBB [31, 32]. In the present study, we found that Dex could inhibit inflammatory factors expression and BBB permeability both *in vivo in vitro* levels, but the effects of Dex were reversed after knocking down CCN1.

As a highly selective α2 adrenergic receptor agonist, Dex has been widely used in clinical surgery anesthesia [33]. Numerous studies have shown that Dex has protective effects on heart, kidney, lung and other organs in ischemia-reperfusion animal models [34]. It has been found that Dex plays a protective role in intestinal ischemia-reperfusion injury through anti inflammation, inhibition of caspase-3 protein expression and apoptosis [35]. It was reported that Dex could attenuate neuronal injury induced by cerebral ischemia-reperfusion by regulating miR-199a [9]. Dex pretreatment alleviated cerebral ischemia/reperfusion injury by inhibiting neuroinflammation through the JAK2/STAT3 pathway [8]. However, the specific targeting molecule has not been unfolded.

CCN1 plays an important role in the complement activation of immune cells. CCN1 can induce integrin dependent activation of monocytes and macrophages in peripheral blood [15, 36]. CCN1 can promote angiogenesis and promote the proliferation and survival of cultured cells [37]. In this study, we demonstrated that the influences of Dex on infarct ratio, Evans blue contents, inflammatory factor levels, tight junction protein, neurological function, and cell apoptosis were reversed by transfection with sh-CCN1. Therefore, we firstly demonstrate that Dex might exert protection effects during MCAO or OGD through targeting CCN1.

In summary, we proved that Dex could remarkably alleviate cerebral ischemia injury by inhibiting BBB breakdown, inflammatory response, and promoting neurological function and tight junction protein expression. In addition, the improvement of cerebral ischemia injury-induced BBB by Dex was achieved by up-regulating CCN1.
